# Endovascular Repair of Spontaneous Rupture of Stent Graft Branch in Thoracoabdominal Aortic Aneurysm—Management, Case Study, and Review

**DOI:** 10.3390/jcm13247687

**Published:** 2024-12-17

**Authors:** Adam Płoński, Adam Filip Płoński, Michał Chlabicz, Jerzy Głowiński

**Affiliations:** Department of Vascular Surgery and Transplantation, Medical University of Bialystok, 15-276 Bialystok, Poland; adamfplonski@yahoo.pl (A.F.P.); chmichal@op.pl (M.C.); jglow@wp.pl (J.G.)

**Keywords:** aneurysm, stent-graft, endovascular therapy, aorta, thoraco-abdominal aortic aneurysm, endovascular surgery

## Abstract

**Background:** Stent-graft implantation is a widely recognized method for endovascular treatment of aortic aneurysms. In cases where the aneurysm involves the thoracic and abdominal aorta, repair including fenestrated and branched stent grafts provides a viable alternative. This approach, initially reserved for patients unsuitable for open surgery, has become preferred for anatomically appropriate thoracoabdominal aortic aneurysms. The Zenith t-Branch system has been extensively studied, demonstrating high technical success rates and acceptable mortality and morbidity. However, complications such as endoleaks, kinking, and stent-graft branch rupture remain significant challenges. **Methods:** We present the case of an 82-year-old male with a thoracoabdominal aortic aneurysm treated with endovascular aneurysm repair using the Zenith t-Branch. Four years post-implantation, he developed a spontaneous rupture of the stent-graft branch, leading to dangerous leakage and aneurysm sac enlargement. An urgent surgical intervention was performed, implanting additional Be-Graft into the damaged branch, restoring stent-graft continuity and revascularizing the superior mesenteric artery. **Results:** The procedure was completed successfully. We conducted a review of the latest literature on endovascular treatment of thoracoabdominal aortic aneurysms with particular emphasis on the possibility of repairing postoperative complications, especially endoleaks. **Conclusions:** While modern technologies have significantly improved outcomes, serious complications persist. Studies emphasize the importance of regular imaging follow-up for early complication detection and management. Continuous advancements in stent-graft technology aim to reduce complications further and improve outcomes. This case underscores the necessity of experienced operators in managing complex and rare complications and highlights the promising future of endovascular techniques in treating thoracoabdominal aortic aneurysms.

## 1. Introduction

Stent-graft implantation represents a globally recognized contemporary approach for endovascular treatment of aortic aneurysms (AA) [1,2,3,4,5]. Over the past 20 years, the availability of endovascular treatments for thoracoabdominal aortic aneurysms (TAAAs) has significantly increased, particularly with the introduction of fenestrated and branched endovascular aortic repair (f/b-EVAR) [6]. Initially, this endovascular method was predominantly reserved for patients who were not suitable candidates for open surgical repair, as noted in the 2017 guidelines by the European Society for Vascular Surgery concerning descending thoracic aortic disease [7]. Recent evidence indicates that f/b-EVAR can lower early mortality and complication rates compared to open repair [8]. However, aortic reinterventions are more common during follow-up after f/b-EVAR, particularly in patients with chronic dissection [8]. These reinterventions predominantly address endoleaks or the instability of the target arteries. With accumulated experience and improvements in both preoperative planning and postoperative care, f/b-EVAR has become the preferred treatment option for anatomically suitable TAAAs in patients without genetically triggered aortic disease or bacterial aortitis [9]. Technological advancements, the aim for reducing patient burden, and the trend towards modern endovascular procedures have all prompted the updating of guidelines based on the latest knowledge and evidence-based medicine [10]. There are various f/b-EVAR endograft systems on the market, mainly divided into custom-made devices (CMDs) tailored for individual patients and off-the-shelf (OTS) stent grafts. Among the latter, the Zenith^®^ t-Branch (Cook Medical, Bloomington, IN, USA) stands out as the most commonly used and studied [6]. Custom-made devices have demonstrated excellent outcomes in both early and long-term scenarios [11,12,13]. However, their use is limited to elective cases due to the lengthy manufacturing process, which can take up to 3 months. For urgent situations, off-the-shelf devices are available immediately, but their suitability is limited by anatomical constraints [14]. The t-Branch, designed with branches for the repair of renal and visceral vessels, is suitable for at least half of all TAAAs [15,16]. Since its commercial launch in Europe in 2012, the t-Branch has been the subject of numerous studies, particularly from highly experienced centers with large patient volumes [17,18,19]. Despite its efficacy, challenges remain, with endoleaks emerging as significant complications. The ideal bridging stent graft remains undefined, with ongoing debate among specialists regarding the use of balloon-expandable versus self-expanding stents [20]. Currently, there is no solid evidence to determine which type of bridging stent graft provides a better outcome following f/b-EVAR [21]. A meta-analysis recently conducted sought to assess how the choice of bridging stent graft affects target vessel (TV) outcomes in the mid-term period following f/b-EVAR. The pooled analysis showed no significant differences in primary patency or freedom from branch-related endoleaks between the stent types over the reported follow-up period (average 17 months, range 12–35 months) [21]. In four studies encompassing 619 target vessels (TV), self-expanding stent grafts (SESG) were associated with fewer secondary interventions and a lower rate of TV instability compared to balloon-expandable stent grafts (BESG) during follow-up [21]. While BESG and SESG demonstrate comparable performance in terms of primary patency and branch-related endoleaks during mid-term follow-up, current retrospective data suggests that SESG may provide better outcomes regarding TV stability and reduced reintervention rates in f/b-EVAR procedures. A recent study investigated how the selection of bridging stents for directional branches (DBs) influences target vessel (TV) outcomes during fenestrated/branched endovascular repair. The findings revealed that DBs reconstructed with BESGs demonstrated reduced freedom from TV instability, TV endoleaks, and TV reinterventions compared to those using SESGs [22]. However, the type of stent construction did not impact DB patency. Since the inception of f/b-EVAR procedures, minimizing the risk of complications associated with the implantation of bridging stent grafts has been a significant challenge, particularly in renal arteries. Renal arteries demand particular attention due to their higher rates of target vessel-related events compared to mesenteric arteries [23]. Respiratory movements pose a problem as they can lead to the potential occlusion of the stent graft. A recently conducted systematic review analyzing data on complications of bridging stent grafts after f/b-EVAR revealed a higher complication rate in renal arteries compared to visceral arteries, at 6% versus 2%, respectively, with an equivalent reintervention rate for visceral target vessels [24]. Leakage at stent-graft junctions stands out as a frequently reported issue in endovascular AA treatment [25]. Complications related to bridging stent grafts in the superior mesenteric artery are rare and are most often associated with stent graft occlusion rather than rupture. Instances of leakage stemming from spontaneous rupture of the thoracoabdominal stent-graft branch, occurring years after implantation and not directly related to invasive intervention, are exceedingly rare yet have potentially catastrophic complications [26]. Currently, there are no guidelines on the ideal technique for connecting target vessels (TV) during the implantation of branched and fenestrated stent grafts, and the existing literature on complications associated with these techniques is extremely limited [24]. At the same time, concerns persist regarding the durability of stent grafts used to bridge aortic grafts to visceral and renal arteries [27]. In our manuscript, we review the latest literature on the complications of endovascular treatment of thoracoabdominal aortic aneurysms and present a highly intricate and very uncommon case involving a patient whose previously implanted stent graft was damaged. Unlike more common intraoperative damage, the stent-graft branch underwent spontaneous destruction, leading to dangerous leakage and the onset of symptoms, thus prompting the decision to undertake an acute intervention.

## 2. Case Presentation

**Figure 1:**
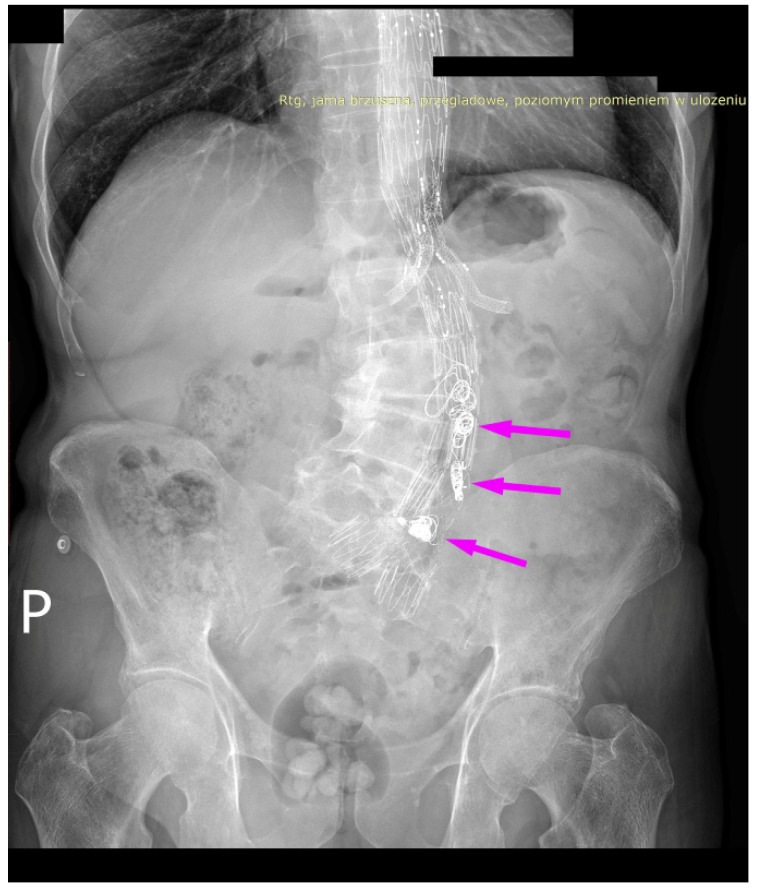
Thoracoabdominal branched stent-graft in the lumen of aorta and visceral arteries. Embolization coils are marked with arrows. Abdominal X-ray.

**Figure 2:**
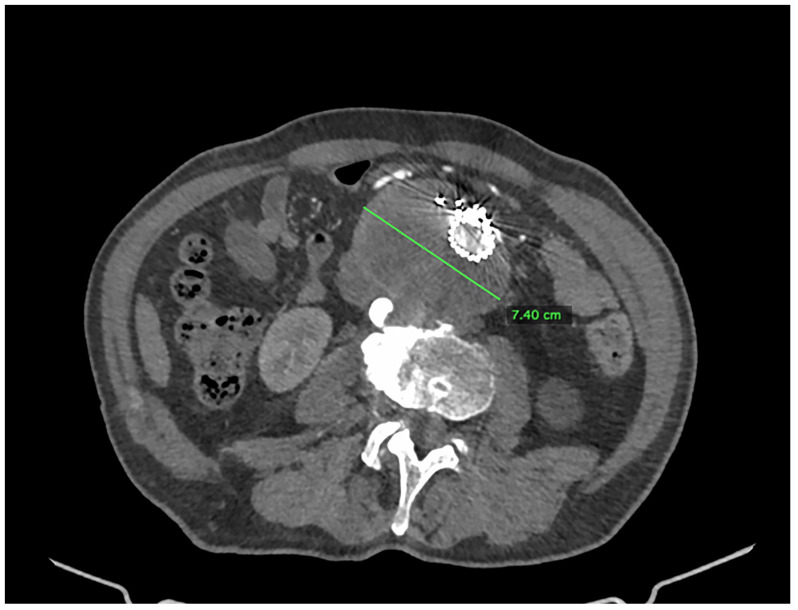
Thoracoabdominal aortic aneurysm with a visible T-Branch stent graft inside. The aneurysm diameter is marked with a green line. Pre-interventional Angio-CT scan performed at the time of the patient’s admission. Axial projection, arterial phase.

**Figure 3:**
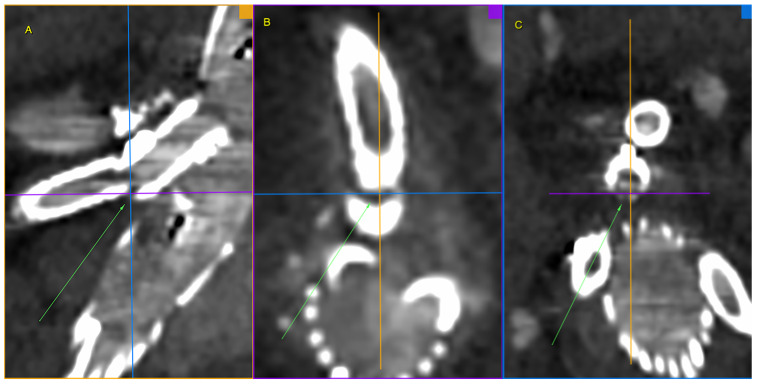
Ruptured superior mesenteric artery branch of stent graft in three planes. The fractured site is centrally located at the intersection of the lines defining the planes and additionally marked with green arrows. Panel (**A**)—sagittal projection, panel (**B**)—axial projection, panel (**C**)—coronal projection. Pre-interventional Angio-CT 3D MPR, arterial phase.

**Figure 4:**
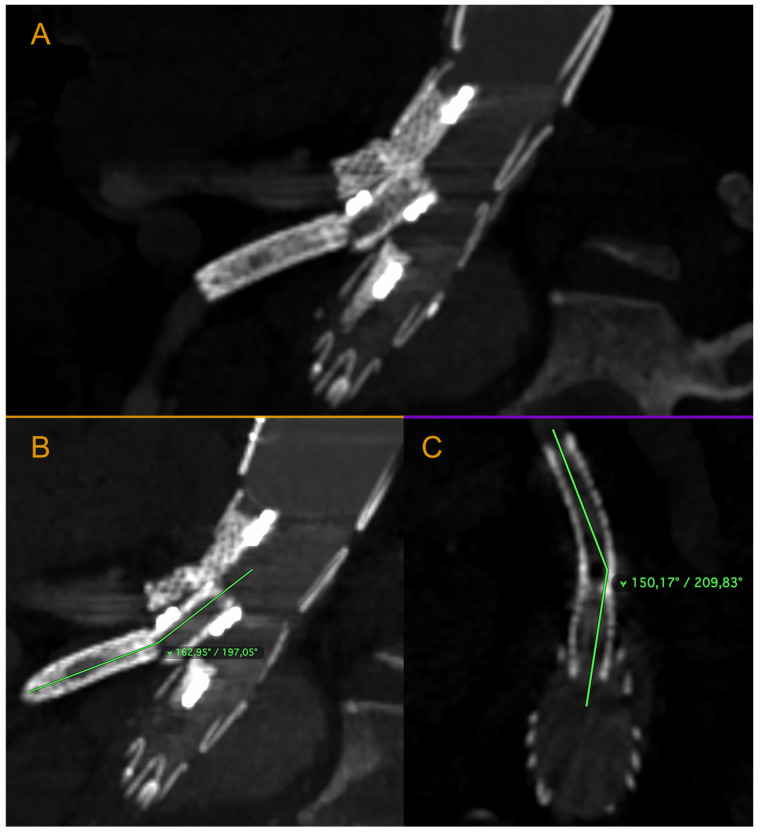
Ruptured superior mesenteric artery branch of stent-graft—the angle between the BeGraft and the vessel. The figure shows a divergence between the axis of the branch and the axis of the superior mesenteric artery. Panel (**A**)—sagittal projection, panel (**B**)—sagittal projection with marked angle, panel (**C**)—axial projection with marked angle. Pre-interventional Angio-CT scans, arterial phase.

**Figure 5:**
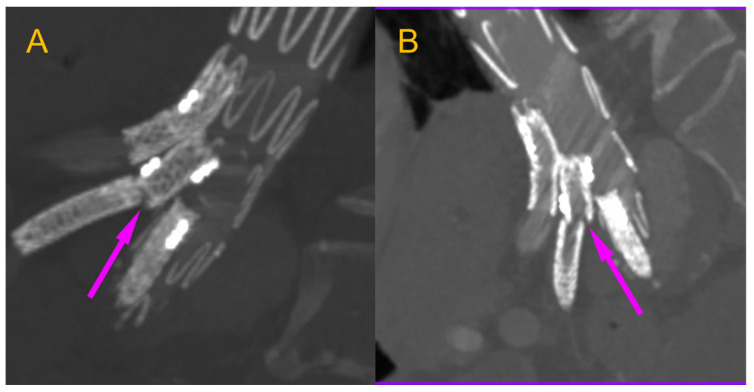
Ruptured superior mesenteric artery branch of stent graft—curved planes. The fractured site is marked with an arrow. Panel (**A**)—first projection, panel (**B**)—second projection. Pre-interventional Angio-CT Curved MPR, arterial phase.

**Figure 6:**
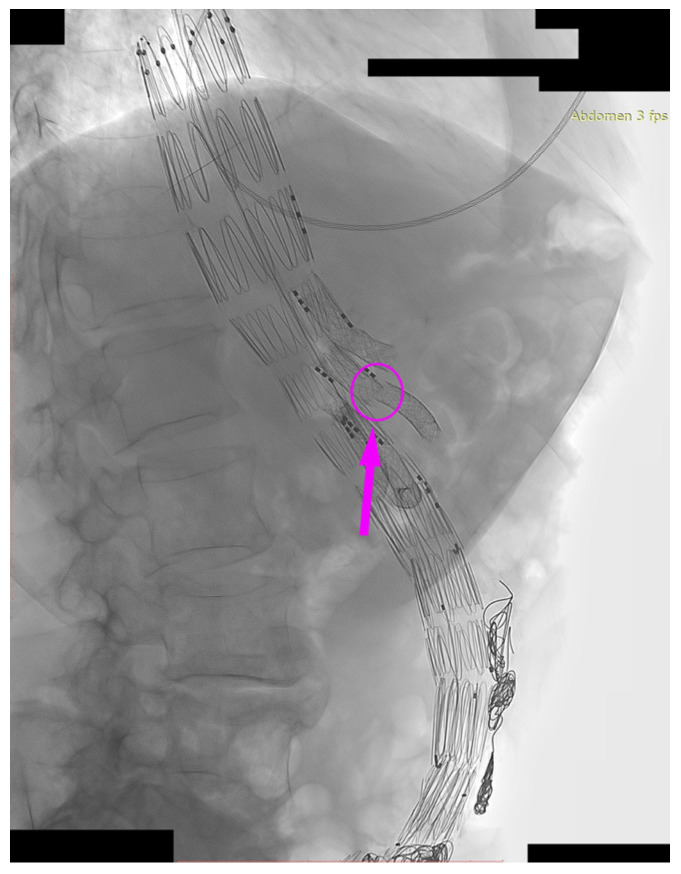
Ruptured superior mesenteric artery branch of stent graft. The fractured site is marked with an arrow. Intraoperative X-ray.

**Figure 7:**
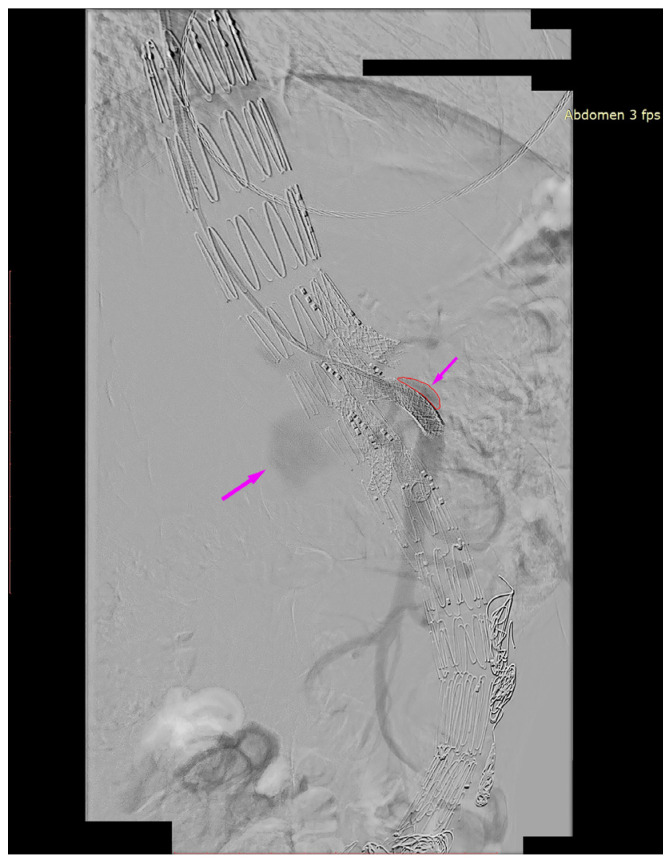
Ruptured superior mesenteric artery branch of stent graft. Leakage outside the stent graft after administration of contrast to the SMA branch. Visible leakage into the aneurysm sac. The leakage is marked with arrows. Intraoperative angiography.

**Figure 8:**
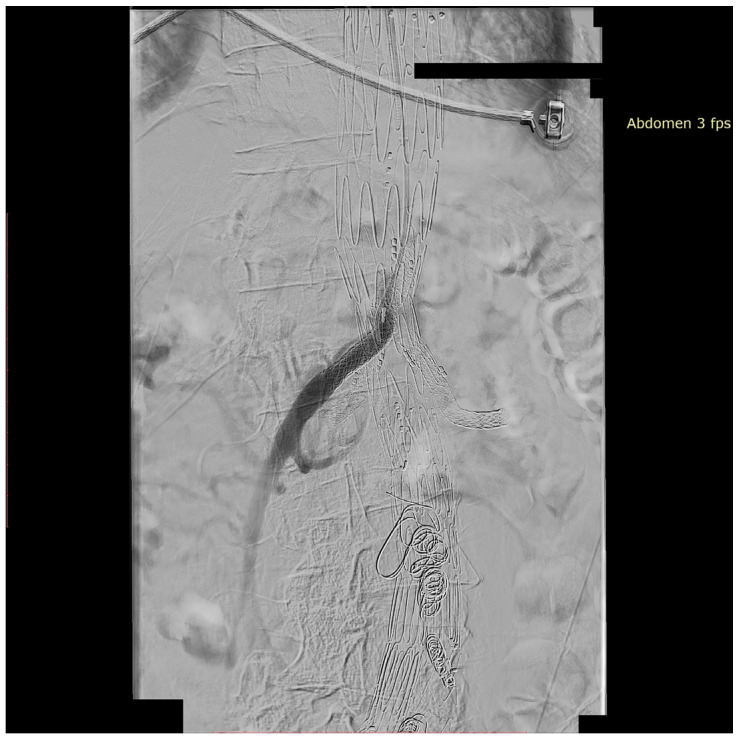
Successful SMA revascularization after BeGraft implantation. No visible contrast leakage. Intraoperative angiography.

**Figure 9:**
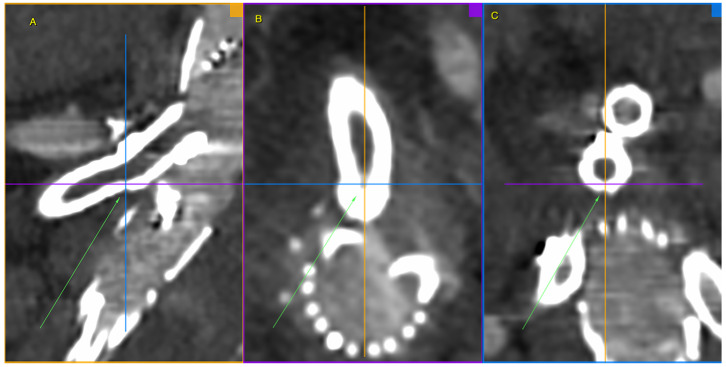
Repaired superior mesenteric artery branch of stent graft in three planes. The mended site is centrally located at the intersection of the lines defining the planes and additionally marked with green arrows. Panel (**A**)—sagittal projection, panel (**B**)—axial projection, panel (**C**)—coronal projection. Control Angio-CT 3D MPR, arterial phase.

**Figure 10:**
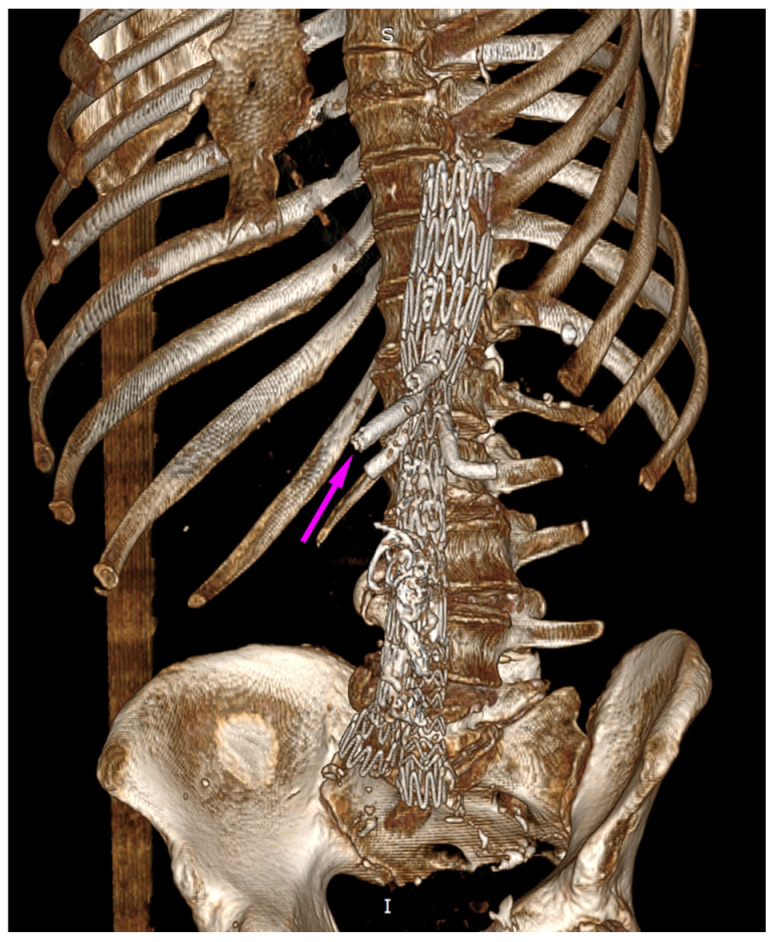
Post-intervention final effect. Newly implanted BeGraft Plus in a previously fractured BeGraft in the SMA. The visible image of the “stent within a stent” is marked with an arrow. Control CT—3D reconstruction.

We present the case of an 82-year-old male patient who appeared in the emergency department due to progressive weakness and abdominal pain (2024). Due to a drop in blood pressure to 80/40 mmHg, a continuous infusion of norepinephrine was initiated. The hemoglobin level was approximately 5.1 g/dL. His medical history included generalized atherosclerosis, renal failure, and a thoracoabdominal aortic aneurysm (TAAA) treated with endovascular aneurysm repair (EVAR) using stent-graft implantation 4 years earlier (2020). At that time, the angio-CT scan revealed an aneurysm starting just below the celiac trunk, extending to the superior mesenteric artery (SMA) and both renal arteries (RAs). The maximum diameter of the aortic aneurysm was 51 mm; however, the left common iliac artery measured 41 mm in diameter, an indication for thoracoabdominal stent graft implantation. The aortic aneurysm itself was completely free of mural thrombus. The patient had also experienced a heart attack 16 years earlier and a transient ischemic attack (TIA) 2 years earlier. Following the stent graft implantation, he underwent multiple successful interventions to address endoleaks. One year after implantation, endovascular treatment with embolization coils was performed to eliminate leakage from the lumbar artery (LA) (2021). Three years post-implantation, he was admitted to address leakage from the inferior mesenteric artery (IMA) using embolization coils as well (Figure 1) (2023). In 2024, an X-ray and CT scan revealed a rupture in a branch of the stent graft located in the SMA in its middle part, along with an enlarged aneurysm sac (74 mm) due to the fracture and leakage (Figure 2, Figure 3, Figure 4 and Figure 5). The angio-CT scan did not reveal any contrast extravasation beyond the vessel wall. However, significant contrast leakage was visible in the area of the visceral arteries within the aneurysm sac. The implanted device was the Zenith t-Branch Thoracoabdominal Endovascular Graft, and the ruptured branch was identified as the Bentley BeGraft (Bentley InnoMed GmbH, Hechingen, Germany). Urgent surgical intervention was performed. Intraoperative angiography (IA) showed a breach in the continuity of the BeGraft material within the SMA, with contrast leakage into the aneurysm sac (Figure 6 and Figure 7). To address the leakage and restore the continuity of the SMA branch, an additional BeGraft Plus 8 × 57 mm was implanted into the damaged branch. Subsequent IA and control CT scans confirmed the elimination of leakage, restoration of stent graft continuity, and revascularization of the SMA (Figure 8, Figure 9 and Figure 10). The surgery was completed without complications, and the patient was discharged home in good general condition. The patient returned for follow-up 3 weeks after the last intervention. An angio-CT examination was performed, revealing no enlargement of the aneurysm sac (74 mm), confirming the elimination of the endoleak, and demonstrating successful revascularization of the SMA. Ongoing follow-up will be continued.

## 3. Discussion

### 3.1. Current State of Knowledge

Currently, f/b-EVAR procedures stand as the preferred approach for TAAA treatment due to their minimal invasiveness and favorable long-term outcomes [28]. Fenestrated and branched endovascular aneurysm repair (F/B-EVAR) has demonstrated high rates of technical and clinical success and low early postoperative mortality [2,29,30,31]. A recent study showed that the use of the Cook t-Branch has proven to be safe and effective in treating complex aortic aneurysms, regardless of the aortic lumen’s caliber [32]. This approach has been shown to be feasible even when maneuverability is reduced, offering low mortality and morbidity rates, as well as acceptable reintervention rates [32]. f/b-EVAR seems to offer a benefit regarding early mortality and perioperative complications compared to open repair [1,29]. While modern technologies and materials have facilitated the treatment of large aneurysms with minimal patient burden, as illustrated by our case, these methods entail the risk of serious complications. Potential complications include endoleak, kinking, fracture, migration, occlusion, and stenosis [33]. This failure mode, in which a branch stent fracture leads to branch vessel perforation, represents a failure not only of the bridging stent (B-Graft) but also of the aortic component (t-Branch). In the largest retrospective analysis to date, including more than 500 consecutively treated patients provided with the t-Branch device for elective and urgent aortic conditions, the authors observed high technical success rates and acceptable levels of mortality and morbidity after 30 days, emphasizing its complementary value for complex endovascular aortic repair [17]. Over time, the combination of progression of vascular disease, endothelial proliferation, continuous arterial movement, and material fatigue may also affect target vessel patency [34]. A propensity score analysis of patients with TAAA undergoing repair suggested an early benefit from endovascular repair compared to open surgery [35]. This advantage is reflected in the composite endpoint, primarily due to reduced 30-day respiratory complications [35]. Despite the absence of explicit guidelines detailing the management of spontaneous rupture of a stent-graft branch, the increasing prevalence of endovascular procedures and the departure from conventional operations suggest a potential rise in such complications. Therefore, recommendations are needed to prevent graft destruction, including more detailed consideration of various anatomical types and improved branch fixation.

### 3.2. Types of Endoleaks and Their Management

Endoleaks have been previously categorized into types 1, 2, 3, 4, and 5. Type 1 endoleaks are further divided into Type 1a and Type 1b, based on their location relative to the stent graft. Type 1a occurs at the proximal seal zone, where the stent graft does not adequately seal against the aortic wall. The primary causes include insufficient overlap, suboptimal graft sizing, or complex aortic anatomy [36]. Management strategies for Type 1a endoleaks involve endovascular techniques aimed at enhancing the proximal seal. These include balloon angioplasty, which involves inflating a balloon at the proximal end of the graft to improve apposition against the aortic wall; extension cuffs, which involve deploying proximal extension cuffs to increase the sealing zone and provide a better fit; and the use of coils or liquid embolic agents to occlude the entry point of the endoleak [37]. Type 1b endoleaks occur at the distal end of the stent graft and are managed similarly. Treatment options include the placement of distal extension grafts to extend the seal zone into a healthier segment of the aorta, balloon angioplasty to improve the seal at the distal end, and embolization techniques.

Type II endoleaks are the most common and arise from retrograde blood flow into the aneurysm sac via collateral vessels, such as the lumbar arteries or the inferior mesenteric artery. In the case presented, we also observed a clear type II endoleak, which had been treated multiple times. Management strategies for this type of endoleak include transarterial embolization of the arteries feeding the endoleak or translumbar embolization, which involves direct puncture of the aneurysm sac through the lumbar route to deliver embolic agents [38]. However, persistent Type II endoleaks can be challenging to manage and may require multiple interventions. In our patient, previous interventions did not reveal any signs of branch rupture. However, it cannot be ruled out that the rupture may have been overlooked. During procedures for treating Type II endoleaks, branch damage can go unnoticed if there is no contrast leakage. It is well understood that we are not always able to eliminate all sources of endoleaks. Our goal is to at least address the major ones, which are potential causes of aneurysm sac enlargement. The coexistence of branch rupture and Type II endoleak is not mutually exclusive and may occur at earlier stages of treatment. To prevent a Type II endoleak, endovascular occlusion of the celiac artery can be performed along with stenting of a thoracoabdominal aortic aneurysm [39]. In a previously conducted study, the celiac artery was successfully occluded in all cases, along with exclusion of the celiac artery aneurysm or thoracoabdominal aortic aneurysm, respectively [39]. The pancreaticoduodenal arteries served as main collateral pathways, but other anastomoses and vascular variations of the celiac artery and its territory were also significant. Advanced techniques, such as embolization via the Riolan arch, can be particularly effective in managing distal leaks. The Riolan arch provides collateral pathways that can be targeted to occlude the endoleak source [40]. One suggested treatment method for endoleaks is transcaval embolization. This technique is a viable alternative for treating type II endoleaks with aneurysm sac enlargement. It can be employed following EVAR for infrarenal abdominal aortic aneurysms, as well as f/b-EVAR for juxtarenal abdominal aortic aneurysms and type IV thoracoabdominal aortic aneurysms [41]. A recent case report detailed an 83-year-old woman with a history of hybrid repair of a thoracoabdominal aortic aneurysm, who presented with aneurysm enlargement attributed to a type II endoleak originating from the celiac artery. Access to the endoleak cavity was achieved via the dorsal pancreatic artery, enabling successful embolization using N-butyl cyanoacrylate and coils [42]. 

Type III endoleak results from fabric tears or component disconnections within the stent graft due to a defect or separation in the stent graft material itself. This type of endoleak necessitates prompt intervention due to its direct communication between the blood flow and the aneurysm sac. This can result from material fatigue, mechanical damage, or poor device integrity. Repair often necessitates the placement of additional stent grafts or relining of the existing graft to seal and bridge the defect [43]. A noteworthy method, especially for challenging endoleaks when other methods are insufficient, is embolization between the aneurysm wall and the stent graft. Utilizing coils or liquid embolic agents to fill the space between the aneurysm wall and the stent graft might effectively seal the leak [44]. Continuous advancement and research in materials have contributed to significant enhancements in stent graft modifications. The primary goal is to further decrease the already low mortality rate and minimize endoleaks. 

Type IV endoleaks are caused by porosity of the graft material allowing blood to seep through the fabric. These are less common. Such leaks often resolve spontaneously as the graft becomes incorporated into the vessel wall. Many Type IV leaks are self-limiting due to thrombus formation and do not require intervention. In persistent cases, the use of stent grafts with additional coatings can mitigate leakages [45]. A type V endoleak (endotension) is characterized by continued aneurysm sac expansion without a visible source of leak on imaging. The management is complex and may involve both imaging to identify the subtle source of the leak and interventions such as graft relining or open surgical conversion. 

### 3.3. Present Possibilities and Future Directions

Innovations such as branched, fenestrated, and physician-modified endografts in the thoracic arch and thoracoabdominal aorta extend the seal zone, thereby reducing the risks associated with proximal and distal endoleaks [46]. Recent research examined the variations in outcomes of f/b-EVAR based on the extent of TAAA [47]. In contrast to open TAAA repair, f/b-EVAR outcomes were comparable for both extensive and nonextensive TAAAs. Variations in perioperative paraparesis, branch instability, and type I or III endoleak likely stemmed from the increased length of aortic coverage and the number of target arteries involved. These findings indicate that high-volume centers conducting f/b-EVAR should anticipate similar outcomes for both extensive and nonextensive TAAA repairs [47].

The ongoing debate regarding the selection of the ideal bridging stent graft remains unresolved. The choice between a self-expanding stent graft (SESG) and a balloon-expandable stent graft (BESG) largely depends on the operator’s subjective decision. Among the self-expanding stent grafts used so far, Fluency and Gore Viabahn have been prominent. Recently, however, few studies have focused on Fluency stent grafts. A notable issue with Gore Viabahn stents is their limited length options—5 cm and 10 cm—lacking intermediate sizes that could have practical applications. A newly introduced stent graft, Solaris, has the potential to address this gap. It offers intermediate lengths of 6 cm and 8 cm, features a user-friendly delivery system, and is considered highly precise. While this system appears promising, few studies have yet been published to evaluate its performance. 

Balloon-expandable stent grafts (BESGs) are convenient to use. A potential alternative to the Bentley BeGraft in the BESG category could be the new iCover stents. Recently published findings from a retrospective single-center study assessing the initial outcomes and long-term durability of the iCover stent graft (iCover-SG) as a bridging stent in fenestrated endovascular aneurysm repair (FEVAR) demonstrated a 94% (82/87) rate of freedom from iCover-SG-related target vessel instability during the study period [48]. The technical success rates were exceptionally high, with primary success achieved in 94% of cases and secondary success in 99% [48]. Another option for BESGs is the GORE VIABAHN VBX. A recently published prospective post-market multicenter registry examined the outcomes associated with VBX stents and reported exceptionally good results. The overall analysis per stent graft reported a primary patency (PP) rate of 95.8% at 1 year, with primary assisted patency (PAP) also at 95.8%, and secondary patency (SP) reaching 97.9% [49]. The analysis per target vessel revealed patency rates as follows: in the celiac trunk, PP, PAP, and SP were all 100%; in the superior mesenteric artery, PP and PAP were 96.0%, with SP at 100%; and in the renal arteries, PP and PAP were 94.2%, with SP at 95.1% [49].

With the increasing incidence of thoracoabdominal aortic pathology such as aneurysms and dissections, along with the growing complexity of treatment options, regular imaging follow-up of patients remains paramount. For patients with thoracoabdominal aortic pathology who have not undergone intervention, vigilant monitoring is essential to detect any changes in aortic size or morphology that could indicate impending rupture or other complications. Patients who have undergone endovascular or open surgical aortic repair should undergo regular follow-up imaging to assess for complications, endoleaks, or other pathologies. Considering their diagnostic accuracy, CT angiography and MR angiography are the preferred imaging modalities for most patients with thoracoabdominal aortic pathology [50]. Given the multifocal nature of thoracoabdominal aortic pathology and its potential complications, imaging of the chest, abdomen, and pelvis is usually necessary for comprehensive evaluation in most patients [50].

Endoleaks may precipitate aneurysm sac enlargement, culminating in rupture [51]. Endovascular aneurysm repair is associated with endoleaks in up to 20% of cases in some series, often necessitating repeat interventions [52]. In a recently presented case, a patient developed a Type II endoleak from the celiac artery, which had not been ligated at its origin, during follow-up after a hybrid TAAA repair [52]. The endoleak was successfully treated with transcatheter coil embolization. A meta-analysis of available studies was conducted, encompassing 197 patients who underwent endovascular repair of thoracoabdominal aortic aneurysms using the t-Branch endograft. The pooled technical success rate was 92.75%, with early endoleaks detected in 10% of cases [53]. Early mortality was reported at 5.8%, and major strokes were observed in 4% of the patients [53]. The aforementioned reports suggest a promising future for the development of modern endovascular techniques. 

### 3.4. Analysis of Management and Patient Summary

The innovative method of implanting a new Be-Graft Plus into the damaged stent graft, which we have proposed and performed, offers significant practical benefits. Currently, there are no studies that clearly identify the cause of unusual complications, such as the dismemberment of stent graft branches. A recent study aimed to assess the influence of target vessel anatomy and bridging stent configuration on target vessel instability in f/b-EVAR. The findings indicated that a vessel diameter smaller than 4 mm and a bridging length exceeding 25 mm were linked to a higher risk of instability in renal target vessels [54]. Additionally, a horizontal misalignment greater than 70 degrees was associated with an elevated risk of complications in visceral target vessels [54]. Complications associated with bridging stent grafts are more commonly due to bending and subsequent occlusion rather than bending followed by stent fracture, which occurs extremely rarely. 

As illustrated in Figure 4, the axis of the branch and the axis of the superior mesenteric artery have become misaligned. The forces exerted as a result of the endoleak may have created an angle and bending, potentially leading to dangerous stent material fracture and further leakage. This is an uncommon case, and it is difficult to find similar descriptions in the literature. It is important to highlight that the event we described occurred suddenly, prompting an urgent decision to implement the appropriate intervention. Throughout the observation period, the patient’s condition remained stable, and he experienced no pain. Endoleak treatment procedures were carried out as planned, and the aneurysm sac enlargement progressed slowly.

However, the most recent intervention we described was an emergency. The patient was admitted to the hospital urgently due to the sudden onset of severe abdominal pain while the patient’s clinical parameters suggested shock. Managing such unusual events requires extensive experience on the part of the operator. The exact cause of the disruption of the bridging stent (B-Graft) in this case remains unclear, though several potential factors may have been involved. Possible contributing factors include excessive angulation of the BeGraft after it exited the sleeve into the SMA during implantation, or excessive stress on the stent graft during balloon expansion. However, the most probable cause of the stent graft branch rupture appears to be the gradual enlargement of the aneurysm sac, driven by persistent and challenging type II endoleaks from the lumbar arteries and IMA. The enlargement of the aneurysm sac could have led to the rupture of the stent graft, which was tightly expanded in the SMA and sleeve through traction. Despite numerous attempts to address the endoleak, no leakage was observed near the visceral vessels in outpatient angio-CT scans or during angiographic studies performed for embolization. However, the emergency angio-CT performed upon the patient’s presentation with severe abdominal pain revealed significant contrast extravasation near the SMA, indicating a severe and sudden complication likely related to progressive aneurysm sac enlargement. Therefore, regular monitoring of patients following EVAR procedures assumes paramount importance, as it facilitates early detection of complications and appropriate intervention [55].

## 4. Conclusions

The development and refinement of technologies, such as the Cook t-Branch device, have improved the treatment of complex aortic aneurysms, offering high technical and clinical success rates, low early postoperative mortality, and acceptable reintervention rates. Despite advancements in EVAR and F/B-EVAR, complications remain a significant concern. Regular imaging follow-up is crucial for the early detection and management of these complications. The presented case underscores the complexity of managing spontaneous complications in endovascular repairs. The sudden rupture of a stent-graft branch and the innovative intraoperative solution highlight the necessity for experienced operators and the importance of immediate decision-making in critical situations. Our case emphasizes the need for ongoing research and monitoring to address rare but severe complications, such as stent-graft branch dismemberment. Future studies should prioritize the design of optimal stent grafts that address the multifaceted requirements for creating a long-lasting f/b-EVAR graft. These include enhanced flexibility, improved trackability, accurate deployment mechanisms, a broader range of diameters and lengths, sufficient radial strength to ensure proper sealing, and resistance to kinking, fractures, and migration [23]. Ultimately, the goal is to determine the best configuration of fenestrations and directional branches tailored to each visceral target, along with developing stent grafts that minimize target-vessel-related events. Managing unusual and severe complications requires substantial expertise on the part of the operator. Given the rarity of complications associated with bridging stent grafts in the superior mesenteric artery, a global registry should be established to document such complications, including the applied repair methods and their short-term and long-term outcomes, supported by meticulously conducted follow-up. The intraoperative decision to implant a new Be-Graft Plus into the damaged branch in this case highlights the critical role of experience and immediate problem-solving abilities in the success of endovascular repair.

## Data Availability

The data presented in this study are available on request from the corresponding author. The data of the patient are not publicly available due to privacy.

## Abbreviations

AA—aortic aneurysm, TAAA—thoracoabdominal aortic aneurysm, EVAR—endovascular aneurysm repair, LA—lumbar artery, IMA—inferior mesenteric artery, SMA—superior mesenteric artery, IA—intraoperative angiography, f/b-EVAR—fenestrated and branched endovascular aortic repair, CMDs—custom-made devices, OTS—off-the-shelf.
